# Injections of Platelet-Rich Plasma: An Emerging Novel Biological Cure for Low Back Pain?

**DOI:** 10.7759/cureus.54048

**Published:** 2024-02-12

**Authors:** Adarsh Jayasoorya, Nitin Samal, Gajanan Pisulkar, Kaustav Datta, Kevin Kawde

**Affiliations:** 1 Department of Orthopaedics, Jawaharlal Nehru Medical College, Datta Meghe Institute of Higher Education and Research, Wardha, IND

**Keywords:** plasma, low back pain, thrombocytes, growth factors, prp injections

## Abstract

Autologous platelet-rich plasma (PRP) injections have emerged as a new biological intervention for many musculoskeletal conditions, such as low back pain (LBP), and have garnered significant attention in recent research endeavors. The recognition of PRP's use is progressively growing; nonetheless, comprehensive clinical validation is required to establish its uses and efficiency. This article offers a thorough evaluation regarding the assurance as well as the efficacy of PRP therapy in the management of low back pain. It specifically focuses on the analysis of clinical trials undertaken in this field.

## Introduction and background

**Figure 1:**
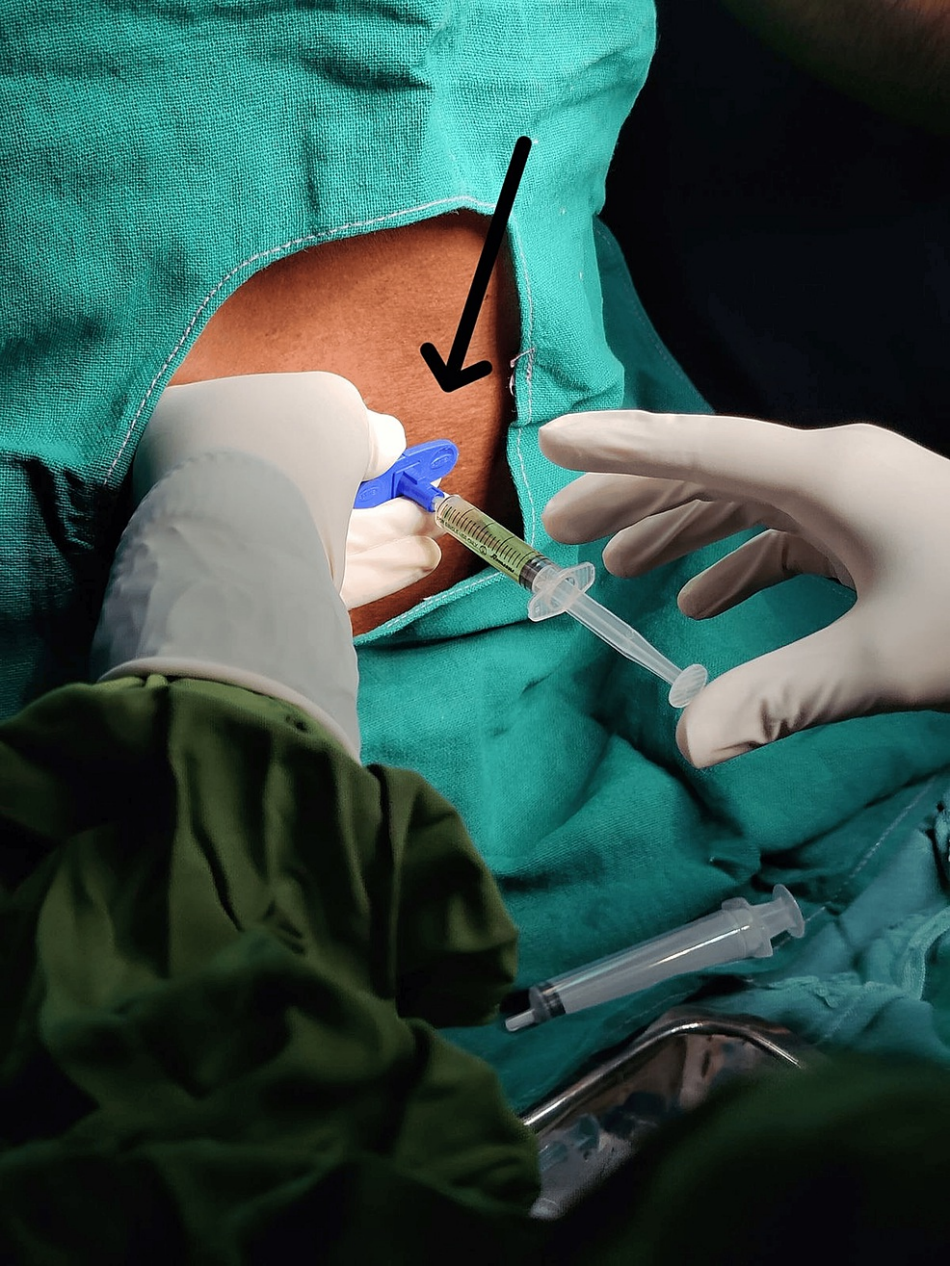
Showing epidural PRP infiltration PRP, platelet-rich plasma

Musculoskeletal disorders are a major cause of disability and a substantial financial burden on society [1]. This illness is more expensive than several other diseases when it comes to both direct as well as indirect costs like reduced productivity and retirement at an early stage. One of the most common musculoskeletal disorders that causes years of healthy life loss due to disability (YLD) in the general community is low back pain (LBP) [1]. Most of the time, there are multiple contributing factors to LBP, including facet joints, spinal ligaments, the vertebral periosteum, the central cord, and spinal nerve roots. Radicular pain symptoms can be caused by pressure on the spinal nerve roots due to compression or inflammation of the spinal nerve roots leaving the spine; this is typically the case when there are spondylotic changes in the spine or disc herniation [2]. The problem at hand is complicated, and its effects on the global economy are enormous. A very common degenerative spinal condition that can cause LBP and radicular limb pain is lumbar disc herniation [3]. Another etiology is intervertebral disc degeneration (IDD), which worsens with age; in individuals over 50, over 80% of intervertebral discs (IVDs) show degeneration-related alterations. Complex fibrocartilaginous tissues called IVDs join neighboring vertebral bodies to allow for spinal movement. It is commonly known that IDD can cause back pain. IVD cells show elevated pro-inflammatory cytokines during IDD. Inflammation and release of pain factors are all primarily brought on by degeneration. Additionally, it leads to the hydrophilic matrix molecules being lost and the extracellular matrix disintegrating, which can result in structural and biomechanical alterations [4]. While facet or sacroiliac joint pathology, disc degeneration, and disc herniation-related pain may be self-limiting, a considerable number of patients may experience chronic pain that necessitates intensive treatment [5]. Minimally invasive techniques can be utilized to manage symptoms and lessen impairment if medication and lifestyle changes do not work [3]. Injection of steroids into the epidural area has been used in the treatment of LPB since the 1950s. Nevertheless, a variety of adverse effects, like insomnia, poor glycemic control, and hypertension, may result from lumbar epidural steroid injections, causing transient problems. As a result, the medical community continues to disagree about the effectiveness of epidural steroid injections, and their brief duration of pain relief further limits their use. Acetaminophen and NSAIDs are the first medications used to treat LBP. NSAIDs are associated with a higher incidence of adverse effects, like stomach ulcers, cardiovascular and renal impairment, even though they have demonstrated greater efficacy than paracetamol [6]. Consequently, new biological therapies are being researched to treat LBP. Platelet-rich plasma (PRP) therapy is one modality that has garnered interest (Figure 1).

Plasma with a strong concentration of platelets which is extracted from a patient's blood is PRP [7]. Because of its increased quantity of platelets, which when activated release different types of multifunctional growth factors (GFs) and are used to repair a variety of avascular tissues, including bones, cartilages, muscles, and tendons; PRP is particularly appealing. It may also offer a new approach to IDD biotherapy [8]. To reduce pain and encourage tissue healing, therapy is a therapeutic strategy that involves injecting concentrated platelets into the surrounding tissue environment [9]. PRP proponents claim that PRP acts as a mediator, bridging the gap between surgical interventions and conservative therapeutic approaches [7].

## Review

Platelet-rich plasma

Recent years had a major increase in desire for PRP, an autologous therapeutic technique, primarily because elite athletes have found success using it for therapeutic purposes [10]. When referring to the procedure of removing and boosting platelets from concentrated plasma for autologous use, the acronym PRP was initially used in the 1970s. Mammals' bone marrow produces platelets, also known as thrombocytes, through the process of megakaryopoiesis. As the main cellular defense mechanism against vascular and tissue damage, macrophages are essential. Innate immune responses, wound healing, angiogenesis, and internal stability (homeostasis) are just a few of the physiological processes in which they actively participate [11]. A healthy person's blood normally contains between 150,000 and 350,000 platelets per microliter [12]. Under normal circumstances, blood samples typically show a composition in which red blood cells (RBCs) make up approximately 94% of the total cellular content, followed by platelets (6%), and white blood cells (1%). To restore the RBC-to-platelet ratio, the primary goal of adding PRP enrichment is to reach a desired composition of 95% platelets and 5% RBCs [11]. The literature currently in publication states that three to nine times the platelet concentration found in blood is required for PRP to demonstrate therapeutic effects [12]. The fraction of PRP that has been enriched is recognized for having higher concentrations of cytokines and GFs, which have been demonstrated to support tissue regeneration and aid in healing. Additionally, it is highly effective in boosting tissue reparative efficacy [11]. When platelets degranulate in response to collagen exposure, GFs are released. Increased cell proliferation and improved cell matrix production are the outcomes of PDGF and TGF-β's roles in cellular remodeling and tissue healing [12]. PRP has primarily been used to treat enthesopathy and chronic tendinopathy, which includes osteoarthritis in the knee. Because of this device's perceived safety, ease of use, and affordability, pain doctors now find it to be of great importance. IVDs, facet joints, ligaments, and radiculopathies have all been treated with spine PRP [10].

Properties and Mechanism of Action of Platelet-Rich Plasma

Activated platelets release a variety of inflammatory chemicals that can be uncomfortable at first but also effectively decrease pain and inflammation [9]. The platelet dynamics of PRP change application that affects the environment before tissue healing and regeneration. These changes occur through several intricate pathways related to stem cell regulation, cellular proliferation, anabolic and catabolic processes, and differentiation. Because of the previously mentioned qualities of PRP, currently, a range of clinical pathological conditions are treated with it including sports injuries that are frequently associated with chronic pain. However, the exact mechanisms underlying these applications are still lacking [9]. The levels of platelet concentration, white blood cell concentration, and activity determine the properties of PRP [13]. It is found that the various PRP-produced factors positively impact cell survival and proliferation. Furthermore, PRP has been demonstrated to inhibit κB pathways and downregulate pro-inflammatory cytokines that are triggered by interleukin-1β and tumor necrosis factor. Moreover, PRP has been shown to support subchondral bone homeostasis and bone mineralization in addition to interacting with endogenous cannabinoid systems [14]. PRP's autologous origin and antimicrobial properties reduce the risk of immunogenic reactions, side effects, and surgical site infection. Numerous important mechanisms were discovered during the current investigation, including the neural regeneration pathway, disc six resorption, and the anti-inflammatory system [13]. PRP can lower the amounts of inflammatory calcitonin gene-related peptides in sensory neurons that innervate the IVDs, according to research by Kamoda H et al. [15]. Thus, by lowering levels of inflammatory neuropeptides, the administration of perineural PRP injections may enhance neural function [15]. In addition, PRP quickens the axonal functional restoration process, which may lessen the likelihood of muscle fibrosis and atrophy and, in the end, restore functionality to the nerve-muscle unit [16].

Preparation of Platelet-Rich Plasma

PRP is extracted from a patient's whole blood sample using a two-step centrifugation procedure that separates the plasma and then concentrates it. Four 8 mL whole blood samples are obtained during the separation procedure and placed into a sterile tube holding an anticoagulant solution made of citrate, phosphate, and dextrose. The tube then experiences centrifugation at a speed of 5,600 rpm for 10 minutes. As a result, a division occurs, yielding the total volume. The platelets that make up the intermediate layer, also known as the buffy coat, emerge in three distinct levels. Leukocytes make up a tiny portion of approximately 5% of the total volume and PPP makes up 40% of the first layer. Finally, RBCs make up the lowest stratum, which makes up 55% of the total volume [17]. At this point, the operator keeps using a sterile syringe to extract all of the PPP and buffy coat before moving them to a different tube devoid of anticoagulant. After that, the sample goes through one more concentration phase, during which centrifugation is applied at a speed of 2,400 revolutions per minute for another 10 minutes. In the second step, platelets are concentrated, which causes different layers to form inside the tube. The previously mentioned layers consist of residual RBC at the bottom, PPP at the top, which makes up approximately 80% of the total volume, and a PRP-based intermediate layer. The next step is to reconstitute platelets in a suitable volume of plasma, usually between 2 mL and 4 mL, to produce PRP. The process of platelet activation is then started by adding a combination of 10% calcium chloride and 10% bovine thrombin, which results in the creation of what is known as "activated" PRP. Because platelets are incorporated into a fibrin mesh, the material has a gelatinous texture that indicates it is ready for use in clinical applications of PRP for LBP [17].

Clinical applications of platelet-rich plasma for low back pain

Experts in medical science have been paying more attention to PRP, and its application in degenerative disc disease management is growing [18]. PRP infiltration into the lumbar region combined with platelet lysate injection into the epidural space represents a new therapeutic approach that can start or accelerate the resorption of herniated lumbar disk material, thereby assisting in the decompression of the affected spinal structures. It is believed that a complex interaction between GFs and cytokines promotes neovascularization and macrophage-induced disk material phagocytosis [19]. A meta-analysis of multiple randomized controlled trials done by Singjie LC et al. found that PRP is more effective than the control group in treating chronic LBP, as demonstrated by the significant decrease in the pain score difference between the PRP and control groups at one, three, and six months after injection [20]. In comparison to controls, PRP injection dramatically improves chronic LBP in the first, third, and sixth months following injection.

Epidural Injections

Bhatia R et al. reported that patients who received autologous PRP epidural injections exhibited appreciable improvements in their ratings on assessment instruments [9]. Throughout the trial's three months, enhancement was continuously maintained and showed no issues. For the treatment of patients with persistent prolapsed IVDs, autologous PRP infiltration may be recommended compared to epidural steroids and surgical procedures. According to research by Singh et al., PRP showed long-lasting relief from intervertebral disc prolapse (IVDP)-induced LBP [21]. Consequently, PRP could be viewed as a safe and promising alternative to steroids and epidural local anesthetics. Ruiz-Lopez et al.'s study examined the therapeutic efficacy and safety of autologous leukocyte-rich (LR)-PRP and corticosteroids for caudal epidural injections under fluoroscopic guidance [22]. The research used a double-blind, randomized design. The outcomes of the study showed that both LR-PRP corticosteroid therapies showed similar levels of safety and effectiveness when used to treat complex chronic lumbar spinal pain. The study also showed that LR-PRP outperformed corticosteroids in terms of enhancing quality of life and delivering more prolonged pain relief. Wongjarupong A et al. reported that at six, 12, and 24 weeks, the visual analog scale of leg pain decreased noticeably and statistically significantly in patients who received PRP injections [13]. Furthermore, a decrease was observed in the Oswestry Disability Index at the 24-week point. The quality of the evidence recommending PRP therapy for LBP has been graded as level II by Machado ES et al. [1]. Centeno C et al.'s study demonstrated that platelet lysate epidural injections have been promised as a treatment for lumbar radicular pain. Notably, following therapy, patients experienced a significant decrease in pain, and this benefit lasted for 24 months [23].

Intrafacet Injections and Intradiscal Injections

Most cases of axial LBP are thought to be caused by lumbar facet joint conditions, with a large percentage of cases being linked to these issues [24]. GFs and cytokines are concentrated where collagen degradation and damage occur inside the disc as a result of PRP being positioned close to the disc. According to one theory, the various GFs and cytokines found in PRP may act as humoral mediators, promoting the body's natural healing processes and potentially reducing LBP [21]. Furthermore, there is a connection between the higher platelet concentration in PRP and the concentration of GFs. In addition, GFs can activate dormant stem cells, which facilitates the process of tissue regeneration, in addition to their inherent healing properties [25]. Akeda et al.'s other study demonstrated that the intradiscal PRP injection produced a significant one even 12 months after the injection [26]. On the other hand, patients with a high number of target lesions or posterior HIZs at the start of the trial were found to be at a higher risk of experiencing unfavorable treatment outcomes. The therapeutic efficacy and safety of autologous PRP for the treatment of lumbar facet syndrome were assessed by Wu J et al. in a study [24]. According to their study's findings, treating patients with lumbar facet joint syndrome with autologous PRP injections for three months has proven to be a novel, safe, and effective method of treating the condition. In contrast to the control group, Tuakli-Wosornu et al. report that after receiving intradiscal PRP treatment, patients' functional rating index (FRI), pain levels as determined by the numerical rating scale (NRS), and overall patient satisfaction ratings improved significantly over eight weeks [27]. Those who received PRP showed consistent and significant improvements in FRI scores throughout the following year's observation period.

Transforaminal Injections

Transforaminal PRP injection guided by ultrasonography has been shown to significantly improve a number of outcomes, including pain relief, nerve regeneration, spinal functionality, and overall quality of life, and the results attained following a one-year follow-up have confirmed that these improvements continue to be effective in the long run. Moreover, Xu Z et al.'s study showed that no issues or adverse effects were noticed during any of the follow-up evaluations [28]. According to this research, PRP injection might be a less risky option for treating lumbar disc herniation. According to a study by Le VT et al., patients' chronic pain can be effectively relieved and their return to work facilitated by using autologous PRP in transforaminal injections [29].

*Platelet-Rich Plasma* *as Regenerative Medicine *

Musculoskeletal injuries have been treated with "regenerative medicine" (RM) since the 1930s. Prolotherapy, PRP therapy, and stem cell therapy are currently used as treatments for musculoskeletal injuries that fall under the umbrella term "RM." Although the ideas behind the different approaches are comparable, the mechanisms behind their reparative abilities are not. Regenerative treatments are making a comeback and becoming more widely used in the musculoskeletal field as possible treatment modalities. Technology has advanced, allowing RM to become PRP. It has been discovered that PRP stimulates the recruitment, proliferation, and differentiation of cells needed for regeneration through a variety of GFs and proteins released from the platelets, which leads to healing [30]. Moreover, platelets can stop anti-inflammatory cytokines like TGF-β from recruiting an excessive number of leukocytes. Because of the biological characteristics of platelets, PRP therapy is regarded as a novel and promising treatment for degenerative changes-related LBP [31]. Therefore, it has been demonstrated that PRP causes inflammatory cells to aggregate by releasing GFs that are mitogenic, angiogenic, and chemotactic from activated platelets [32]. Given that PRP administration does not require sophisticated equipment or specialized training to perform, its advantages are associated with a cost advantage. Furthermore, worries about disease transmission or immunogenic reactions can be ignored because of their primarily autologous origin. Therefore, in the context of wound healing and tissue regeneration, platelet-enriched materials have gained significant relevance over the past 10 years and are increasingly the subject of clinical and experimental research [33].

Complications and limitations of platelet-rich plasma therapy 

Although they are all extremely rare, puncture-related and drug-related complications were the most common PRP injection complications. PRP injection side effects included post-injection soreness and stiffness, chest pain, dyspnea, jitteriness, and pain on the opposite side [34]. Results may vary depending on PRP preparation techniques, activator kinds, pathology types to be treated, administration routes and timings, and PRP's interaction with other therapies [35]. Second, there is a lack of standardization and heterogeneity in PRP preparations. Nonetheless, a PRP product that is sold commercially can be utilized [32].

## Conclusions

For those with LBP, autologous PRP spinal injections have demonstrated efficacy and safety as a conservative treatment option. Furthermore, compared to steroids, autologous PRP is a better therapeutic option due to its longer-lasting effectiveness, according to the research. Numerous studies have provided evidence regarding the safety and potential short- and long-term effects of platelet-rich products and PRP in the treatment of LBP. In-depth double-blind randomized trials have to be a part of future research projects to validate PRP therapy as a treatment backed by empirical data. Strict guidelines for patient selection should be followed in these studies to guarantee consistency in the PRP components utilized.
